# Traditional Medicinal Uses and Ethnopharmacological Significance of *Alchemilla* L. Species in Azerbaijan

**DOI:** 10.3390/plants15142241

**Published:** 2026-07-22

**Authors:** Javanshir Isayev, Elvin Nagiyev, Gunay Jafarova, Nilufar Safarova, Vagida Mammadova

**Affiliations:** Department of Pharmacognosy, Azerbaijan Medical University, Baku AZ1022, Azerbaijan; isayev.cavanshir@amu.edu.az (J.I.); en.naghiyev@gmail.com (E.N.); nilupharm@gmail.com (N.S.); mvagida70@mail.ru (V.M.)

**Keywords:** *Alchemilla*, Azerbaijan, ethnopharmacology, medicinal plants, Rosaceae, traditional medicine

## Abstract

The present ethnopharmacological study investigated the traditional medicinal uses of *Alchemilla* species distributed across different geobotanical regions of Azerbaijan. Field surveys were conducted in five mountainous regions of the country during 2024–2025, and ethnopharmacological data were collected from 125 informants through semi-structured interviews and questionnaire surveys. A total of 21 *Alchemilla* species belonging to the Rosaceae family were documented. Quantitative ethnobotanical indices, including Use Value (UV), Relative Frequency of Citation (RFC), Fidelity Level (FL), and Informant Consensus Factor (ICF), were applied to evaluate the ethnobotanical significance of the recorded species. The results revealed that *Alchemilla* species are mainly used for the treatment of digestive, hepatobiliary, respiratory, and skin disorders, as well as for the management of diabetes. The highest ICF value was recorded for antipyretic use (ICF = 1.00), whereas the greatest number of use reports was documented for digestive system disorders (Nur = 278). *Alchemilla sericea*, *A. sericata*, *A. caucasica* and *A. grossheimii* exhibited the highest UV and RFC values. The high quantitative ethnobotanical indices reflect the relative local importance of these species and the strong consensus among informants regarding their traditional medicinal uses. The findings demonstrate the important role of *Alchemilla* species in the traditional medicine of Azerbaijan and indicate that species with high ethnobotanical indices represent promising candidates for future phytochemical and pharmacological investigations.

## 1. Introduction

The Republic of Azerbaijan, located within the Caucasus floristic region, harbors more than 5000 vascular plant species, representing approximately 70% of the Caucasian flora. Its flora comprises over 160 plant families and more than 1100 genera, reflecting the country’s remarkable plant diversity [1,2]. In recent years, increasing scientific attention has been directed toward the pharmacognostic, phytochemical, and ethnopharmacological investigation of medicinal plants occurring in the country. Numerous ethnobotanical and ethnopharmacological studies conducted across different regions of Azerbaijan have documented the traditional uses of medicinal plants, providing valuable insights into local ethnomedical knowledge and plant-based healthcare practices [3,4,5,6,7,8,9].

**Figure 1:**
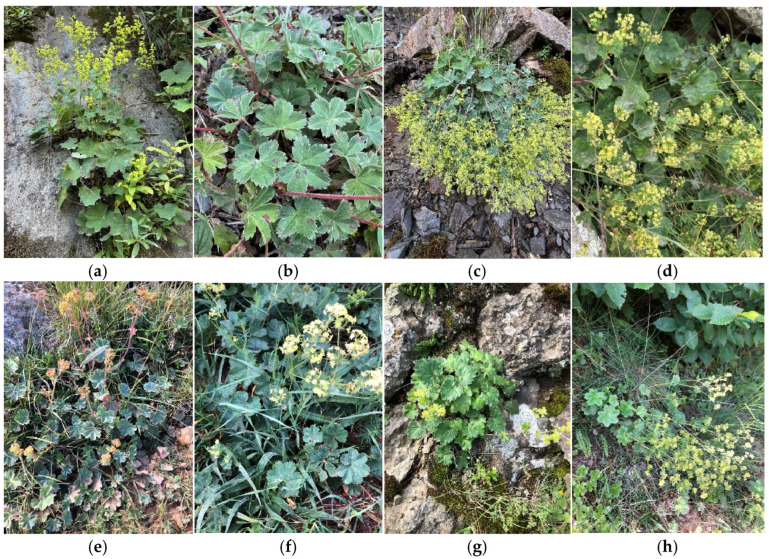
Representative *Alchemilla* species documented during the present ethnopharmacological survey in Azerbaijan: (**a**) *A. sericea*; (**b**) *A. retinervis*; (**c**) *A. diversipes*; (**d**) *A. caucasica*; (**e**) *A. persica*; (**f**) *A. raddeana*; (**g**) *A. sericata*; (**h**) *A. hyrcana*.

Among the species-rich plant families of Azerbaijan, Rosaceae Juss. is represented by 29 genera and approximately 200 species, while globally the family comprises more than 100 genera and over 3000 species [1,2]. Many Rosaceae species possess medicinal, nutritional, and ornamental value and are widely used in both traditional and scientific medicine owing to their rich content of biologically active compounds. Among the genera of the family Rosaceae, *Alchemilla* L. has attracted considerable scientific interest owing to its taxonomic diversity, medicinal importance, and long-standing use in traditional medicine. Species of *Alchemilla* are predominantly perennial herbaceous plants that are widely distributed in mountainous and foothill regions. The flora of Azerbaijan includes 21 species of this genus. Among them, *A. hyrcana* (Bus.) Juz. and *A. jaroschenkoi* Grossh. are endemic to Azerbaijan, whereas *A. raddeana* (Bus.) Juz. is considered an endemic species of the Caucasus region. Species of *Alchemilla* occur mainly in the alpine and subalpine meadows of the Greater and Lesser Caucasus, as well as the Talysh Mountains, and are also found in rocky and stony habitats and along the banks of mountain rivers and lakes [1]. Representative *Alchemilla* species documented during the present study are presented in Figure 1.

The illustrated *Alchemilla* species represent part of the morphological diversity recorded during the survey, providing a visual overview of the medicinal taxa documented in the present study.

Phytochemical and pharmacological studies of *Alchemilla* species have demonstrated that these plants are rich sources of flavonoids, tannins, phenolic compounds, polysaccharides, essential oils, and other biologically active constituents [10,11]. Species belonging to this genus have been reported to exhibit antioxidant, anti-inflammatory, antibacterial, antiviral, and wound-healing activities [12,13]. In particular, *A. vulgaris* L. has been included in the European Pharmacopoeia and is currently used as a component of various medicinal preparations [14].

In recent years, growing interest in medicinal plants and traditional medicine has stimulated numerous ethnobotanical and ethnopharmacological studies worldwide. These investigations have documented medicinal plants used by local communities, their preparation methods and therapeutic applications, including the traditional uses of *Alchemilla* species, thereby contributing to the preservation and advancement of ethnomedical knowledge [15,16,17,18,19,20].

Despite the widespread distribution of *Alchemilla* species in Azerbaijan, their ethnopharmacological significance remains insufficiently explored. Most species of this genus occurring in the country’s flora have not yet been subjected to comprehensive pharmacognostic and ethnopharmacological investigations. Given the increasing global interest in medicinal plants and the importance of preserving traditional knowledge, documenting the folk medicinal uses of *Alchemilla* species may provide valuable information for future pharmacognostic, phytochemical, and pharmacological studies.

The aim of the present study was to document the traditional medicinal uses of *Alchemilla* species in Azerbaijan through interviews with local informants, to systematize the recorded ethnopharmacological knowledge, and to assess their potential significance as medicinal plant resources for future pharmacognostic and phytochemical research. The findings are expected to contribute to the preservation of traditional knowledge, the sustainable utilization of local plant resources, and the identification of promising taxa for future scientific investigation.

## 2. Results

### 2.1. Demographic Characteristics of the Respondents

The ethnopharmacological survey was conducted during 2024–2025 in five different geobotanical regions of Azerbaijan. A total of 125 respondents were interviewed during the study. The demographic characteristics of the respondents are presented in Table 1. Respondents were selected using the snowball sampling method.

Of the respondents participating in the survey, 82 were male (65.60%) and 43 were female (34.40%). The distribution of respondents across age groups was as follows: 4 individuals (3.20%) were aged 28–40 years, 15 (12.00%) were aged 41–50 years, 21 (16.80%) were aged 51–60 years, 26 (20.80%) were aged 61–70 years, 33 (26.40%) were aged 71–80 years, and 26 (20.80%) were aged 81–92 years. The predominance of informants aged 61–92 years indicates that traditional ethnobotanical knowledge is primarily preserved among the older generation.

Regarding educational attainment, 67 respondents (53.60%) had completed secondary education, 55 (44.00%) had higher education, and 3 (2.40%) had incomplete secondary education. With respect to marital status, 83 respondents (66.40%) were married, 7 (5.60%) were single, and 35 (28.00%) were widowed.

The survey included individuals representing a variety of occupational groups. Among the respondents, 36 were teachers, 28 were retired, 14 were farmers, 13 were municipal representatives, 11 were local administrative representatives, 9 were housewives, 8 were foresters, and 6 were shepherds. In addition, interviews were conducted with eight local plant experts possessing specialized knowledge of medicinal plants.

### 2.2. Diversity and Distribution of Alchemilla Species

The ethnopharmacological survey documented a total of 21 *Alchemilla* species used by local communities across different geobotanical regions of Azerbaijan. These species were found to be distributed predominantly within the Greater and Lesser Caucasus mountain systems, as well as in the Talysh region [1,2].

Among the documented species, *A. caucasica* Bus., *A. diversipes* Juz., *A. grossheimii* Juz., *A. persica* Rothm., and *A. sericea* Willd. were reported to be used for the treatment of four different disease categories. *A. raddeana* (Bus.) Juz., *A. sericata* Reichenb. ex Bus., and *A. smirnovii* Juz. were associated with three disease categories. Eight species were used in two disease categories, whereas six species were associated with only one disease category. The ethnopharmacological uses and quantitative indices of *Alchemilla* species are presented in Table 2.

**Figure 2:**
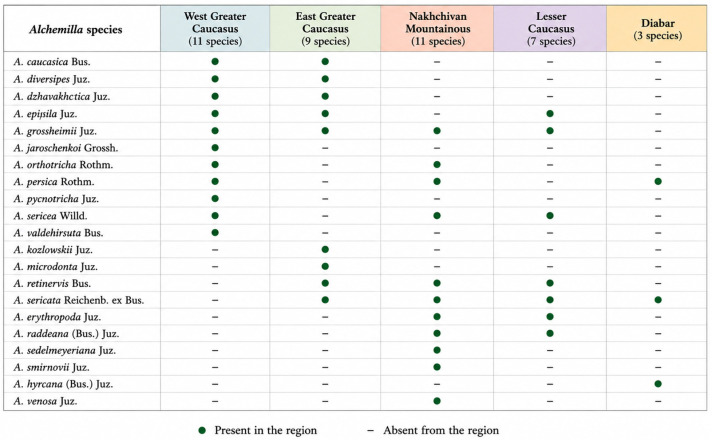
Distribution of the documented *Alchemilla* species across the five geobotanical regions of Azerbaijan. Green circles indicate the presence of a species in a region, whereas dashes indicate its absence.

The regional distribution of the documented *Alchemilla* species across the five geobotanical regions of Azerbaijan is presented in Figure 2. The West Greater Caucasus and Nakhchivan exhibited the highest species richness, with 11 recorded species each, followed by the East Greater Caucasus (9 species) and the Lesser Caucasus (7 species), whereas Diabar had the lowest species richness with only three recorded species.

### 2.3. Disease Categories and Ethnomedicinal Uses

The ethnopharmacological survey revealed that *Alchemilla* species are traditionally used for the treatment of disorders affecting the oral cavity, respiratory system, cardiovascular system, digestive system, urinary system, liver, and skin. In addition, these species were reported to be used for gynecological disorders and diabetes mellitus.

Based on the collected data, the reported traditional medicinal uses were classified into different disease categories according to the International Classification of Diseases, 11th Revision (ICD-11) [21]. The results showed that *Alchemilla* species were most frequently used for digestive system disorders, with 11 species accounting for a total of 278 use reports. Hepatobiliary disorders were represented by 95 use reports, followed by respiratory system disorders with 84 use reports. Disorders of the oral cavity accounted for 76 use reports.

The Informant Consensus Factor (ICF) values ranged from 0.91 to 1.00. The highest ICF value was recorded for the category of fever-related conditions (ICF = 1.00). High levels of informant consensus were also observed for infectious and parasitic diseases (ICF = 0.97), digestive system disorders (ICF = 0.96), hepatobiliary disorders (ICF = 0.96), gynecological disorders (ICF = 0.96), and urinary system disorders (ICF = 0.96). The ICF value was 0.95 for respiratory system disorders, skin diseases and wound healing, and oral diseases. Cardiovascular diseases showed an ICF value of 0.93, whereas the lowest ICF value was recorded for endocrine disorders (ICF = 0.91) (Table 3).

To further investigate whether the therapeutic use categories differed among the five geobotanical regions of Azerbaijan, the distribution of citation reports was compared across regions. As shown in Table 4, digestive system disorders represented the predominant therapeutic category in all five regions, with the highest number of citation reports recorded in the Lesser Caucasus (109 reports) and Nakhchivan (97 reports). In contrast, fever-related conditions were reported only in Nakhchivan, whereas infectious and parasitic diseases accounted for comparatively few citation reports across all regions.

Overall, the Lesser Caucasus region exhibited the highest number of citation reports (335), followed by Nakhchivan (251) and the West Greater Caucasus (249). In contrast, Diabar recorded the lowest number of citation reports (73), indicating a comparatively narrower diversity of documented traditional medicinal uses of *Alchemilla* species.

Pearson’s chi-square analysis demonstrated a significant association between therapeutic categories and geobotanical regions (χ^2^(40) = 333.39, *p* < 0.001) (Table 5). The calculated Cramér’s V value (0.280) indicated a moderate association, suggesting that the distribution of therapeutic use categories varied among the five geobotanical regions of Azerbaijan.

### 2.4. Plant Parts Used and Methods of Preparation

The results of the study indicated that different plant parts of *Alchemilla* species were used in traditional medicine. The herb (aerial parts, including stems, leaves, and flowers) was the most frequently utilized plant material, accounting for 57.1% of all recorded uses. In addition, rhizomes (28.6%), leaves (9.5%), and flowers (4.8%) were also used for the treatment of various ailments. In the majority of the investigated species, the use of aerial parts predominated.

According to the respondents, *Alchemilla* species were primarily prepared as infusions (57%), decoctions (34%), or administered with food (9%). Infusion and decoction were the predominant methods of preparation, whereas administration with food was less frequently reported. The choice of preparation method appeared to be influenced by the plant part used, with rhizomes being commonly prepared as decoctions, whereas aerial parts, leaves, and flowers were more frequently used for the preparation of infusions. The mode of administration varied according to the therapeutic application. Preparations intended for gastrointestinal disorders, liver disorders, diabetes mellitus, and respiratory disorders were administered orally, whereas remedies used for wound healing and dermatological disorders were applied topically. Preparations used for oral ulcers and gingival inflammation were commonly employed as mouth rinses.

**Figure 3:**
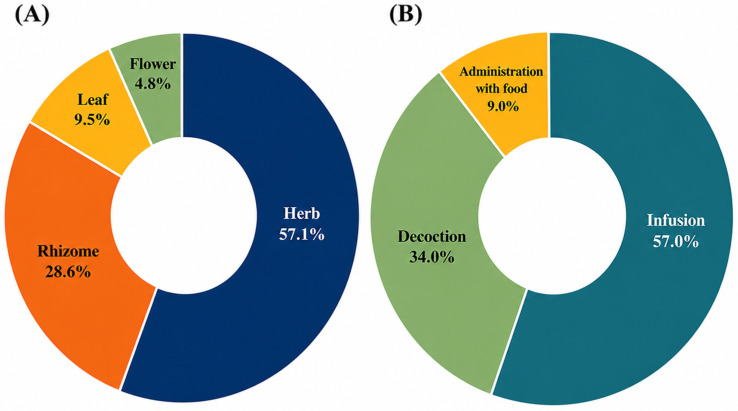
Distribution of plant parts used (**A**) and methods of preparation (**B**) of *Alchemilla* species used in traditional medicine.

The distribution of plant parts used (A) and methods of preparation (B) is presented in Figure 3.

### 2.5. Analysis of Use Value (UV) and Relative Frequency of Citation (RFC)

The quantitative ethnobotanical analysis revealed that the highest Relative Frequency of Citation (RFC) was recorded for *A. sericea* Willd. (RFC = 0.54). This species was primarily used for the treatment of gynecological disorders, gastrointestinal disorders, diabetes mellitus, and dermatological disorders. High RFC values were also observed for *A. sericata* Reichenb. ex Bus. (RFC = 0.50), *A. caucasica* Bus. (RFC = 0.44), and *A. grossheimii* Juz. (RFC = 0.39).

The highest Use Value (UV)was likewise recorded for *A. sericea* Willd. (UV = 0.70). In addition, *A. sericata* Reichenb. ex Bus. (UV = 0.56), *A. caucasica* Bus. (UV = 0.49), and *A. grossheimii* Juz. (UV = 0.47) also exhibited high UVs.

### 2.6. Analysis of Fidelity Level (FL%) Values

According to the Fidelity Level (FL%) analysis, several *Alchemilla* species exhibited a high degree of specificity for the treatment of particular disease categories. *A. dzhavakhetica* Juz., *A. jaroschenkoi* Grossh., *A. kozlowskii* Juz., *A. microdonta* Juz., *A. orthotricha* Rothm., and *A. retinervis* Bus. showed an FL value of 100%. These species were reported by informants exclusively for the treatment of a single disease category.

High FL values were also recorded for *A. epipsila* Juz. (73.33%), *A. sedelmeyeriana* Juz. (71.79%), *A. erythropoda* Juz. (61.11%), and *A. pycnotricha* Juz. (52.94%). Species with the highest FL values were primarily used for digestive, hepatic, urinary, and dermatological disorders. Detailed FL% values of the documented *Alchemilla* species are presented in Table 2.

## 3. Discussion

The present ethnopharmacological study demonstrated that *Alchemilla* species occupy an important place in the traditional medicinal practices of local communities across different geobotanical regions of Azerbaijan. The documentation of 21 species used for the treatment of various disease categories highlights both the wide recognition of this genus among local populations and its significance within traditional medical knowledge. The predominance of uses related to digestive system disorders, hepatobiliary disorders, and respiratory system disorders suggests that *Alchemilla* species have long been regarded as valuable medicinal resources for the management of these health conditions. Similar findings have been reported in ethnobotanical studies conducted in Turkey and Lithuania, where digestive and respiratory disorders were among the most frequently treated disease categories using medicinal plants [17,19,20].

The high Informant Consensus Factor (ICF) values obtained in the present study indicate a strong level of agreement among respondents regarding the medicinal uses of *Alchemilla* species. In ethnopharmacological research, high ICF values are generally considered indicative of well-established traditional knowledge and consistent patterns of plant use within local communities. In particular, the high consensus observed for digestive system disorders and hepatobiliary disorders suggests a long-standing and stable tradition of using *Alchemilla* species for the treatment of these conditions. Although direct comparisons should be interpreted cautiously because most previous studies evaluated all medicinal plants rather than a single genus, the high consensus values recorded in the present study further support the cultural importance of *Alchemilla* species in Azerbaijani traditional medicine.

In addition to the high informant consensus, statistical analysis demonstrated significant regional variation in the distribution of therapeutic use categories across the five geobotanical regions of Azerbaijan. The predominance of digestive system disorders in all regions, together with the observed differences in the frequencies of other therapeutic categories, suggests that the traditional medicinal uses of *Alchemilla* species are influenced by regional ecological conditions and the preservation of local ethnomedicinal knowledge.

The widespread use of *Alchemilla* species in traditional medicine may be associated with their rich phytochemical composition. Previous studies have demonstrated that species of this genus contain flavonoids, phenolic compounds, tannins, anthocyanins, coumarins, triterpenes, and other metabolites possessing antioxidant properties. In particular, the use of tannin-rich *Alchemilla* species for gastrointestinal disorders, inflammatory conditions, and wound healing is consistent with the biological activities reported in phytochemical and pharmacological studies. Furthermore, antioxidant, anti-inflammatory, antimicrobial, wound-healing, antidiabetic, and hepatoprotective activities have been documented for several *Alchemilla* species, providing a scientific basis for some of their traditional medicinal applications [16].

In the present study, quantitative ethnobotanical indices highlighted differences in the cultural importance of the recorded *Alchemilla* species. The quantitative ethnobotanical analysis revealed that *A. sericea*, *A. sericata*, *A. caucasica*, and *A. grossheimii* exhibited the highest UV and RFC values. These findings indicate that these species are among the most widely recognized and frequently used medicinal plants within local communities. The particularly high UV and RFC values recorded for *A. sericea* may be attributed to its use in the treatment of multiple disease categories, highlighting its importance in traditional medicinal practices. Among these four species, *A. caucasica* is the only species for which phytochemical characterization and experimental pharmacological evidence have been reported, including gastroprotective activity in an experimental animal model and the identification of several flavonoids, such as quercetin-3-O-glucuronide, apigenin, and catechin [22]. In contrast, published phytochemical and pharmacological information on *A. sericea*, *A. sericata*, and *A. grossheimii* remains very limited. To the best of our knowledge, no clinical studies involving these four species have been reported to date. Furthermore, species with high FL% values demonstrated a greater degree of specificity for particular therapeutic applications, suggesting that they may represent promising candidates for future phytochemical and pharmacological investigations.

The use of *Alchemilla* species for respiratory disorders observed in the present study is also supported by ethnobotanical observations from neighbouring regions, where *A. crinita* was reported to be used for asthma, bronchitis, and cough. Similarly, several *Alchemilla* species recorded in the present study were used for respiratory disorders, including cough and other respiratory complaints, indicating a certain degree of similarity in the traditional use of this genus across different regions [19].

The frequent use of aerial parts (herb) documented in the present study is consistent with previous phytochemical investigations demonstrating that *Alchemilla* species are rich in tannins, flavonoids, phenolic acids, and other polyphenolic compounds, which represent the major bioactive constituents of the genus. Phytochemical and pharmacological studies of different *Alchemilla* plant parts and their extracts have associated these compounds with antioxidant, anti-inflammatory, antimicrobial, gastroprotective, wound-healing, hepatoprotective, and antidiabetic activities [16]. These findings provide a plausible phytochemical basis for several of the traditional medicinal applications documented in the present study, particularly those related to gastrointestinal disorders, liver disorders, wound healing, respiratory disorders, and diabetes mellitus. Although phytochemical evidence is currently available for only a limited number of *Alchemilla* species, the similar traditional uses documented for many Azerbaijani taxa suggest that further phytochemical investigations may reveal comparable bioactive constituents. Nevertheless, experimental pharmacological evidence remains unavailable for most Azerbaijani *Alchemilla* species, and therefore their traditional medicinal uses require further phytochemical characterization and pharmacological validation.

The predominance of herb as the most frequently used plant material and the widespread use of infusions and decoctions indicate that these preparation methods remain deeply rooted in traditional medicinal practices. Similar patterns of plant-part utilization and remedy preparation have been documented in ethnobotanical studies from different geographical regions, including Lithuania and Turkey, where aerial parts, leaves, and flowers were among the most frequently utilized plant materials and infusion and decoction represented the principal methods of preparation [17,20]. The preference for aerial plant parts may be explained by their greater accessibility, ease of collection, and practical use in the preparation of traditional remedies.

Interviews conducted with respondents in the study areas indicated that concerns regarding the collection and conservation of wild medicinal plants have increased in recent years. The expansion of human activities and the intensive use of mountainous areas as grazing lands may contribute to the reduction in natural habitats and populations of numerous wild plant species. These pressures also affect species of the genus *Alchemilla*. In particular, intensive grazing in summer pasture areas may exert significant anthropogenic pressure on natural populations of these plants.

Among the species documented in the present study, *A. hyrcana* is included in the Red Book of the Republic of Azerbaijan, whereas *A. grossheimii* has been listed in the Pink List [23]. The conservation and sustainable use of the natural populations of these species are therefore of particular importance. During awareness-raising discussions with local communities, special emphasis was placed on the sustainable use and conservation of medicinal plant resources. These observations highlight the need for continued conservation efforts and the promotion of sustainable harvesting practices to ensure the long-term preservation of both medicinal plant diversity and associated traditional knowledge. In addition, the cultivation of medicinally important *Alchemilla* species could represent a sustainable conservation strategy by reducing harvesting pressure on natural populations while ensuring a reliable source of plant material for future phytochemical and pharmacological research.

The present study has certain limitations. Information regarding the dosage, treatment duration, and possible adverse effects of the recorded herbal remedies was not systematically collected during the ethnopharmacological interviews. Therefore, these aspects could not be evaluated in the present study. Future ethnopharmacological investigations should incorporate these parameters to provide a more comprehensive assessment of the traditional medicinal use and safety of *Alchemilla* species.

Overall, the present study demonstrated that *Alchemilla* species occupy an important place in the traditional medicinal practices of Azerbaijan. The high use reports, strong informant consensus, and elevated quantitative ethnobotanical indices highlight the cultural and medicinal significance of this genus. These findings provide valuable ethnobotanical evidence for prioritizing *Alchemilla* species in future phytochemical and pharmacological research. By identifying the species that are most frequently used and show the highest informant consensus, the present study provides a rational basis for selecting priority taxa for bioactivity screening, phytochemical characterization, and experimental pharmacological validation. Furthermore, the findings emphasize the importance of detailed studies aimed at identifying and characterizing the bioactive compounds responsible for the traditional medicinal uses of these species.

## 4. Materials and Methods

### 4.1. Study Area

The study was conducted in five different geobotanical regions of the Republic of Azerbaijan, including the western and eastern parts of the Greater Caucasus, the Lesser Caucasus, the Nakhchivan mountainous region, and the Talysh mountain system [1,2]. The study sites were primarily located in lower and middle mountain zones at elevations ranging from 900 to 2900 m above sea level (a.s.l.). These regions are characterized by high floristic diversity and diverse mountain ecosystems.

**Figure 4:**
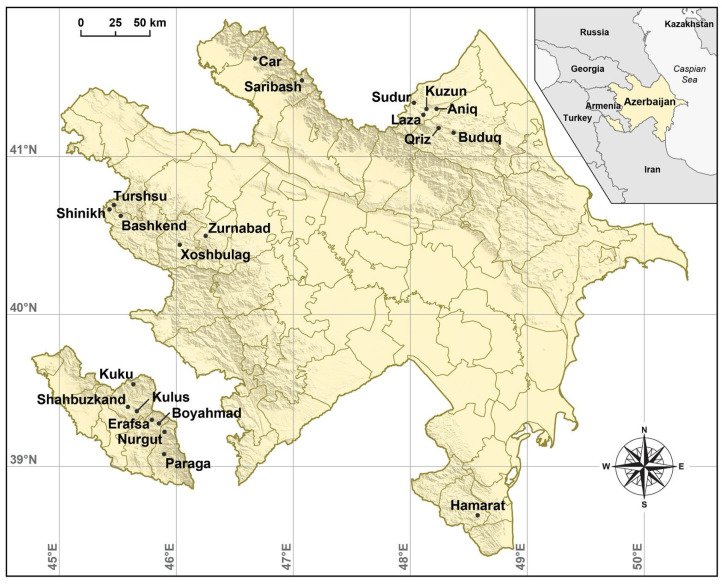
Geobotanical regions of Azerbaijan and the location of the study areas included in the present ethnopharmacological survey.

Most of the surveyed areas were characterized by mountainous terrain and semi-arid climatic conditions. The winter season generally extends from November to April, while temperatures in some high-mountain areas remain below the freezing point for prolonged periods during winter. The geographical distribution of the study areas is presented in Figure 4.

### 4.2. Ethnopharmacological Data Collection

Ethnopharmacological data were collected during 2024–2025 through structured questionnaires and face-to-face interviews conducted among rural communities. The structured questionnaire used in the ethnopharmacological survey is provided in Supplementary File S1. A total of 125 respondents were interviewed across the five geobotanical regions included in the study. Respondents were selected using the snowball sampling method, whereby each participant recommended individuals possessing traditional ethnobotanical knowledge for subsequent interviews.

Eligible participants were local residents aged 18 years or older who voluntarily agreed to participate in the study. Individuals were selected based on their knowledge and experience regarding the traditional use of medicinal plants. The initial key informant was identified through recommendations from local community members as a person recognized for extensive traditional knowledge, and subsequent participants were recruited through participant referrals. Individuals who reported no knowledge of the traditional use of medicinal plants were excluded from the study.

Interviews were conducted in residential settlements, agricultural fields, forested areas, and high-mountain meadows. In addition, surveys were carried out among shepherds and their family members residing in summer pasture areas.

Prior to data collection, permission was obtained from local administrative representatives, municipal authorities, and community elders. Before each interview, the objectives of the study were explained to the participants, and verbal informed consent was obtained. Participation was voluntary, and all ethnopharmacological information was recorded anonymously. The study was conducted in accordance with local ethical standards and followed the principles of the International Society of Ethnobiology (ISE) Code of Ethics [24], with due respect for the traditional knowledge, cultural values, and customary practices of the local communities.

### 4.3. Plant Collection and Identification

Plant specimens were collected from different geobotanical regions of Azerbaijan during the period from March to November in 2024–2025. The collected specimens were identified using materials from the Herbarium Collection of the Institute of Botany, Ministry of Science and Education of the Republic of Azerbaijan, and the Plants of the World Online (POWO) database [25]. Voucher specimens were prepared, assigned voucher numbers, and deposited in the Department of Pharmacognosy, Azerbaijan Medical University.

Information on collection localities, geographical coordinates, elevation, and voucher numbers of the investigated *Alchemilla* species is provided in Table 6.

### 4.4. Quantitative Ethnobotanical Analysis

To quantitatively evaluate the collected ethnopharmacological data, the Use Value (UV), Frequency of Citation (FC), Relative Frequency of Citation (RFC), Fidelity Level (FL%), and Informant Consensus Factor (ICF) indices were calculated.

Quantitative data analysis

Quantitative ethnobotanical indices were used to evaluate the relative importance of documented *Alchemilla* species and to assess patterns of traditional medicinal use among local communities. The collected ethnopharmacological data were analyzed using the following indices:

Use value (UV)

The Use Value (UV) index was calculated to evaluate the relative importance of each *Alchemilla* species based on the frequency of its reported medicinal uses [9,26].

UV = ΣUi/N

where ΣUi represents the total number of use reports recorded for a given species, and N denotes the total number of respondents participating in the survey. Higher UVs indicate species that are more frequently cited and have greater significance in local traditional medicinal practices.

Frequency of citation (FC)

Frequency of Citation (FC) refers to the number of respondents who reported the use of a particular *Alchemilla* species [27,28]. This index reflects the extent to which a species is recognized and utilized within the study area. Higher FC values indicate that a species is known and cited by a larger proportion of informants.

Relative Frequency of Citation (RFC)

The Relative Frequency of Citation (RFC) index was used to evaluate the local importance and recognition of each *Alchemilla* species among respondents [4,29]. The index was calculated using the following formula:

RFC = FC/N

where FC represents the number of respondents who mentioned a particular species and N is the total number of respondents included in the survey. RFC values range from 0 to 1, with higher values indicating greater recognition and use of a species within the local community.

Fidelity Level (FL %)

The Fidelity Level (FL%) was calculated to determine the degree of specificity with which a particular *Alchemilla* species was used for the treatment of a given disease category [30]. The index was estimated using the following formula:

FL (%) = (Np/N) × 100

where Np represents the number of respondents who cited a species for a specific disease category, and N denotes the total number of respondents who reported the use of that species for any medicinal purpose. Higher FL% values indicate a stronger consensus regarding the use of a species for a particular therapeutic application.

Informant Consensus Factor (ICF)

The Informant Consensus Factor (ICF) was used to assess the degree of agreement among respondents regarding the use of *Alchemilla* species for different disease categories [31]. The index was calculated according to the following formula:

ICF = (Nur − Nt)/(Nur − 1)

where Nur represents the total number of use reports recorded for a particular disease category and Nt denotes the number of species used to treat that category. Higher ICF values indicate a greater level of consensus among respondents concerning the medicinal use of plants for specific health conditions.

### 4.5. Statistical Analysis

To further evaluate regional variation in the traditional medicinal uses of *Alchemilla* species, citation reports (use reports) were classified into 11 therapeutic categories according to the International Classification of Diseases, 11th Revision (ICD-11). A contingency table was constructed using therapeutic categories and the five geobotanical regions of Azerbaijan. Pearson’s chi-square (χ^2^) test of independence was performed to assess whether the distribution of citation reports differed significantly among regions. The strength of the association was evaluated using Cramér’s V. Statistical significance was accepted at *p* < 0.05.

## 5. Conclusions

This study represents the first comprehensive ethnopharmacological investigation of *Alchemilla* species in Azerbaijan and documents the traditional medicinal uses of 21 species distributed across different geobotanical regions of the country. Quantitative ethnobotanical analyses demonstrated that *Alchemilla* species occupy an important place in the traditional medicinal practices of Azerbaijan, with the highest use reports and informant consensus recorded for digestive and hepatobiliary disorders. Among the documented taxa, *A. sericea*, *A. sericata*, *A. caucasica*, and *A. grossheimii* exhibited the highest UV and RFC values, indicating their prominent cultural importance and widespread traditional use.

The present findings provide valuable ethnopharmacological evidence supporting the preservation of traditional medicinal knowledge and contribute to the identification of priority *Alchemilla* species for future scientific research. The documented patterns of traditional use, together with the available phytochemical and pharmacological evidence, suggest that these species represent promising candidates for bioactivity screening, phytochemical characterization, and experimental pharmacological validation. Furthermore, the sustainable conservation of wild *Alchemilla* populations remains essential for preserving both the biological diversity of the genus and the associated traditional medicinal knowledge for future generations.

## Figures and Tables

**Table 1:** Demographic Characteristics of the Respondents.

Characteristics	*n*	%
Gender		
Female	43	34.4
Male	82	65.6
Age group (years)		
28–40	4	3.2
41–50	15	12
51–60	21	16.8
61–70	26	20.8
71–80	33	26.4
81–92	26	20.8
Education level		
Secondary education	67	53.6
Incomplete secondary education	3	2.4
Higher education	55	44
Marital status		
Married	83	66.4
Single	7	5.6
Widowed	35	28
Occupation		
Housewife	9	7.2
Teacher	36	28.8
Local administrative representative	11	8.8
Municipal representative	13	10.4
Retired	28	22.4
Forester	8	6.4
Shepherd	6	4.8
Farmer	14	11.2
Herbalist	8	6.4

**Table 2:** Ethnopharmacological Uses and Quantitative Indices of Documented *Alchemilla* Species in Azerbaijan.

Plant Species	Vernacular Name	Ailments Treated	Informant Citation	FL (%)	FC	Plant Part Used	RFC	ΣU	UV
*A. caucasica* Bus.	Qafqaz şaxduranı	Oral ulcers	21	34.43	55	Herb	0.44	61	0.49
		Liver disorders	15	24.59					
		Atherosclerosis	6	9.84					
		Gastrointestinal disorders	19	31.15					
*A. diversipes* Juz.	Müxtəlif yarpaqlı şaxduran	Wound-healing agent	7	12.73	29	Rhizome	0.23	55	0.44
		Atherosclerosis	9	16.36					
		Oral ulcers	17	30.91					
		Antitussive	22	40					
*A. dzhavakhetica* Juz.	Cavaxetiya şaxduranı	Anthelmintic	18	100	18	Leaf	0.14	18	0.14
*A. epipsila* Juz.	Epipsila şaxduranı	Liver disorders	22	73.33	24	Flower	0.19	30	0.24
		Kidney disorders	8	26.67					
*A. erythropoda* Juz.	Qırmızı-saplaqlı şaxduran	Oral ulcers	7	38.89	15	Herb	0.12	18	0.14
		Dermatological disorders	11	61.11					
*A. grossheimii* Juz.	Qrossheym şaxduranı	Liver disorders	14	23.73	49	Herb	0.39	59	0.47
		Gastric ulcer	21	35.59					
		Common cold	18	30.51					
		Gynecological disorders	6	10.17					
*A. hyrcana* (Bus.) Juz.	Hirkan şaxduranı	Kidney disorders	20	52.63	27	Herb	0.22	38	0.3
		Gastrointestinal disorders	18	47.37					
*A. jaroschenkoi* Grossh.	Yaroşenko şaxduranı	Gastrointestinal disorders	23	100	23	Herb	0.18	23	0.18
*A. kozlowskii* Juz.	Kozlovski şaxduranı	Arterial hypertension	17	100	17	Rhizome	0.14	17	0.14
*A. microdonta* Juz.	Xırdadişli şaxduran	Gastrointestinal disorders	14	100	14	Herb	0.11	14	0.11
*A. orthotricha* Rothm.	Düz-tüklü şaxduran	Oral ulcers	23	100	23	Rhizome	0.18	23	0.18
*A. persica* Rothm.	Fars şaxduranı	Common cold	12	22.64	47	Herb	0.38	53	0.42
		Gastrointestinal disorders	15	28.3					
		Diabetes mellitus	7	13.21					
		Gynecological disorders	19	35.85					
*A. pycnotricha* Juz.	Sıxtüklü şaxduran	Diuretic	18	52.94	24	Herb	0.19	34	0.27
		Liver disorders	16	47.06		Rhizome			
*A. raddeana* (Bus.) Juz.	Radde şaxduranı	Wound-healing agent	21	45.65	29	Herb	0.23	46	0.37
		Broncholytic	17	36.96					
		Gingival inflammation	8	17.39					
*A. retinervis* Bus.	Torlu damarlı şaxduran	Gastric ulcer	24	100	24	Rhizome	0.19	24	0.19
*A. sedelmeyeriana* Juz.	Sedelmayer şaxduranı	Antipyretic	11	28.21	32	Herb	0.26	39	0.31
		Choleretic	28	71.79					
*A. sericea* Willd.	Zərfi tüklü şaxduran, ipəkvari şaxduran	Gynecological disorders	24	27.27	67	Herb	0.54	88	0.7
		Gastrointestinal disorders	27	30.68					
		Diabetes mellitus	11	12.5					
		Dermatological disorders	26	29.55					
*A. sericata* Reichenb. ex Bus.	İpəkli şaxduran	Anthelmintic	19	27.14	62	Leaf	0.5	70	0.56
		Gastrointestinal disorders	37	52.86					
		Atherosclerosis	14	20					
*A. smirnovii* Juz.	Smirnov şaxduranı	Diabetes mellitus	9	21.95	26	Herb	0.21	41	0.33
		Arterial hypertension	12	29.27					
		Gastrointestinal disorders	20	48.78					
*A. valdehirsuta* Bus.	Çox tüklü şaxduran	Broncholytic	15	46.88	24	Rhizome	0.19	32	0.26
		Wound-healing agent	17	53.12					
*A. venosa* Juz.	Damarlı şaxduran	Diabetes mellitus	7	14.58	43	Herb	0.34	48	0.38
		Gastrointestinal disorders	41	85.42					

**Table 3:** Disease Categories and Informant Consensus Factor (ICF) Values of Documented *Alchemilla* Species.

Disease/Use Category	NT	Nur (Use Reports)	ICF
Digestive system disorders	11	278	0.96
Hepatobiliary disorders	5	95	0.96
Respiratory system disorders	5	84	0.95
Skin diseases and wound healing	5	82	0.95
Oral diseases	5	76	0.95
Cardiovascular diseases	5	58	0.93
Gynecological disorders	3	49	0.96
Urinary system disorders	3	46	0.96
Endocrine disorders	4	34	0.91
Infectious and parasitic diseases (Anthelmintic)	2	37	0.97
Fever-related conditions	1	11	1

**Table 4:** Distribution of citation reports among therapeutic categories across the five geobotanical regions of Azerbaijan.

Therapeutic Category	West Greater Caucasus	East Greater Caucasus	Nakhchivan	Lesser Caucasus	Diabar
Cardiovascular diseases	6	30	16	14	3
Digestive system disorders	51	53	97	109	32
Endocrine disorders	6	0	22	11	2
Fever-related conditions	0	0	11	0	0
Gynecological disorders	20	2	13	30	7
Hepatobiliary disorders	33	19	30	36	0
Infectious and parasitic diseases	8	16	4	19	5
Oral diseases	37	15	18	15	0
Respiratory system disorders	35	16	16	35	4
Skin diseases and wound healing	32	3	24	58	0
Urinary system disorders	21	3	0	8	20
Total citation reports	249	157	251	335	73

Citation reports were classified into therapeutic categories according to the ICD-11 framework.

**Table 5:** Results of Pearson’s chi-square analysis comparing therapeutic categories among the five geobotanical regions of Azerbaijan.

Statistical Parameter	Value
Pearson’s chi-square (χ^2^)	333.39
Degrees of freedom (df)	40
*p*-value	<0.001
Cramér’s V	0.280

Statistical significance was accepted at *p* < 0.05.

**Table 6:** Collection localities, geographical coordinates, elevation, and voucher numbers of *Alchemilla* Species.

Plant Species	Collection Locality	Geographical Coordinates	Elevation (m a.s.l.)	Voucher Number
*A. caucasica* Bus.	Laza village, Qusar District	41°17′42″ N, 48°06′22″ E	1950	AZE-IJ-001
*A. diversipes* Juz.	Qriz village, Quba District	41°12′56″ N, 48°14′48″ E	2450	AZE-IJ-002
*A. dzhavakhetica* Juz.	Kuzun village, Qusar District	41°20′27″ N, 48°08′09″ E	1450	AZE-IJ-003
*A. epipsila* Juz.	Bashkend village, Gadabay District	40°38′46″ N, 45°30′56″ E	1550	AZE-IJ-004
*A. erythropoda* Juz.	Zurnaabad village, Goygol District	40°30′22″ N, 46°14′25″ E	1500	AZE-IJ-005
*A. grossheimii* Juz.	Shahbuzkand village, Shahbuz District	39°22′43″ N, 45°34′45″ E	1650	AZE-IJ-006
*A. hyrcana* (Bus.) Juz.	Hamarat village, Lerik District	38°38′50″ N, 48°34′45″ E	1400	AZE-IJ-007
*A. jaroschenkoi* Grossh.	Sanab village, Aghsu District	41°31′27″ N, 47°04′30″ E	1600	AZE-IJ-008
*A. kozlowskii* Juz.	Car village, Zagatala District	41°40′19″ N, 46°41′14″ E	900	AZE-IJ-009
*A. microdonta* Juz.	Aniq village, Qusar District	41°19′58″ N, 48°13′16″ E	1350	AZE-IJ-010
*A. orthotricha* Rothm.	Shinikh village, Gadabay District	40°41′15″ N, 45°25′44″ E	1500	AZE-IJ-011
*A. persica* Rothm.	Kulus village, Shahbuz District	39°21′43″ N, 45°39′38″ E	1700	AZE-IJ-012
*A. pycnotricha* Juz.	Sudur Village, Qusar District	41°22′40″ N, 48°02′08″ E	1787	AZE-IJ-013
*A. raddeana* (Bus.) Juz.	Xoshbulag village, Dashkasan District	40°27′45″ N, 46°01′37″ E	1750	AZE-IJ-014
*A. retinervis* Bus.	Turshsu village, Gadabay District	40°42′00″ N, 45°28′00″ E	1400	AZE-IJ-015
*A. sedelmeyeriana* Juz.	Nurgut village, Ordubad District	39°13′30″ N, 45°53′54″ E	1900	AZE-IJ-016
*A. sericea* Willd.	Erafsa village, Julfa District	39°17′31″ N, 45°47′05″ E	1700	AZE-IJ-017
*A. sericata* Reichenb. ex Bus.	Boyahmad village, Julfa District	39°16′03″ N, 45°50′45″ E	2900	AZE-IJ-018
*A. smirnovii* Juz.	Paraga village, Ordubad District	39°04′30″ N, 45°53′36″ E	1650	AZE-IJ-019
*A. valdehirsuta* Bus.	Buduq village, Quba District	41°11′10″ N, 48°22′07″ E	1800	AZE-IJ-020
*A. venosa* Juz.	Kuku village, Shahbuz District	39°31′24″ N, 45°37′21″ E	1550	AZE-IJ-021

## Data Availability

All data generated or analyzed during this study are included in this published article.

## Supplementary Materials

The following supporting information can be downloaded at: https://www.mdpi.com/article/10.3390/plants15142241/s1. Supplementary File S1: Structured questionnaire used in the ethnopharmacological survey.

## Abbreviations

Abbreviation	Meaning
UV	Use Value
FC	Frequency of Citation
RFC	Relative Frequency of Citation
FL	Fidelity Level
ICF	Informant Consensus Factor
ICD-11	International Classification of Diseases 11th Revision
