# Supplementary material for: Traditional Medicinal Uses and Ethnopharmacological Significance of Alchemilla L. Species in Azerbaijan
Source: Plants (Basel). 2026 Jul 22;15(14):2241. doi: 10.3390/plants15142241 (PMC13417494; doi:10.3390/plants15142241)
Supplement: Supplementary file 1 [file plants-15-02241-s001.zip › plants-4401117-supplementary.pdf]

Supplementary File S1: Structured questionnaire used in the ethnopharmacological survey.

Medicinal Plant Questionnaire

AZƏRBAYCAN TİBB UNİVERSİTETİ

Location (Village): ___________________________

Date: ____ / ____ / ________

This questionnaire is intended to document the traditional use of medicinal plants in your village. Participation is voluntary, and all information provided will be used solely for scientific research purposes.

1. PARTİCİPANT INFORMATİON

1. Age: ___________________

2. Sex: ☐ Female ☐ Male

3. Education level: ☐ Secondary ☐ Higher ☐ Other ___________________

4. How many years have you lived in this village? ___________________ years

2. INFORMATİON ON MEDİCİNAL PLANTS

5. Which medicinal plants do you know?

____

____

6. For which health conditions do you use these plants?

☐ Common cold

☐ Fever

☐ Cough

☐ Expectorant

☐ Gastrointestinal disorders

☐ Gastric ulcer

☐ Flatulence

☐ Laxative

☐ Hypertension

☐ Atherosclerosis

☐ Hemorrhoids

☐ Arrhythmia

☐ Sleep disorders

☐ Sedative

☐ Kidney disorders

☐ Diuretic

☐ Liver disorders

☐ Choleretic

☐ Oral ulcers

☐ Wound healing

☐ Diabetes mellitus

☐ Anti-inflammatory

☐ Skin diseases

☐ Toothache

☐ Other: __________________

7. Which plant part do you use?

☐ Leaf ☐ Flower ☐ Root ☐ Fruit ☐ Herb (aerial parts)

8. How do you prepare the remedy?

☐ Infusion

☐ Dry and store

☐ Juice

☐ Decoction

☐ Use fresh

☐ Other: __________________

9. Where do you collect the plant?

☐ Meadow

☐ Mountain

☐ Purchased from the market

☐ Forest

☐ Home garden

10. From whom did you learn about these medicinal plants?

☐ Parents
☐ Grandparents
☐ Local community members knowledgeable about medicinal plants
☐ Self-taught

11. In your opinion, are these medicinal plants still used as frequently as in the past?

☐ Yes, they are used as before.
☐ No, they are used less frequently than before.
☐ They are used more frequently than before.
☐ I do not know.

Additional comments:

____

____

____
